# Supplementary material for: Microbiological culture versus 16S/18S rRNA gene PCR-sanger sequencing for infectious keratitis: a three-arm, diagnostic cross-sectional study
Source: Front Med (Lausanne). 2024 Aug 12;11:1393832. doi: 10.3389/fmed.2024.1393832 (PMC11352289; doi:10.3389/fmed.2024.1393832)
Supplement: Supplementary file 1 [file Table_1.docx]

Supplementary Table 1. A 2x2 cross-tabulation for calculating sensitivity and specificity.

|  | | **Disease status (based on reference standard)** | |
| --- | --- | --- | --- |
|  |  | Yes | No |
| **Index test** | Positive | True positive (TP) | False positive (FP) |
|  | Negative | False negative (FN) | True negative (TN) |
| **Sensitivity and specificity** | | Sensitivity:  $=\frac{TP}{TP+FN}$ | Specificity:  $=\frac{TN}{FP+TN}$ |
